# Advanced Degradation and Remediation Strategies for Per- and Polyfluoroalkyl Substances (PFASs): Challenges and Future Perspectives

**DOI:** 10.3390/toxics14060499

**Published:** 2026-06-07

**Authors:** Xiaohui Zhang, Tongshun Han, Xiaofeng Yao, Rui Zhao, Wenjun Sun, Liang Pei, Jianguo Zhao, Peigao Duan

**Affiliations:** 1 Engineering Research Center of Coal-Based Ecological Carbon Sequestration Technology of the Ministry of Education, Key Laboratory of Graphene Forestry Application of National Forest and Grass Administration, Shanxi Datong University, Datong 037009, China; baixue215@163.com (X.Z.); 16632816406@163.com (T.H.); zhaorui@163.com (R.Z.); jianguozhao9150@163.com (J.Z.); 2 Shanxi Provincial Ecological Environment Monitoring and Emergency Support Center, Shanxi Provincial Academy of Ecological Environment Sciences, Taiyuan 030024, China; sofya1974@163.com; 3 School of Environment, Tsinghua University, Beijing 100084, China; wsun@tsinghua.edu.cn; 4 Xinjiang Institute of Ecology and Geography, Chinese Academy of Sciences, Urumqi 830011, China; 5 College of Resources and Environment, University of Chinese Academy of Sciences, Beijing 100049, China; 6 School of Chemical Engineering and Technology, Xi’an Jiaotong University, Xi’an 710049, China; pgduan@xjtu.edu.cn

**Keywords:** per- and polyfluoroalkyl substances (PFAS), defluorination, transformation-product toxicity, integrated separation–destruction treatment trains

## Abstract

Per- and polyfluoroalkyl substances (PFASs) are persistent aquatic contaminants whose strong C–F bonds make conventional water treatment ineffective. This review critically synthesizes recent progress in aqueous PFAS degradation through four mechanistic routes: oxidation-driven, biodegradation, reduction-driven, and nonradical processes. Rather than evaluating technologies by parent-compound disappearance alone, we compare their defluorination and mineralization capacities, matrix tolerance, byproduct risks, energy demand, operational stability, and technology readiness. Oxidative and reductive systems can promote rapid degradation or defluorination, but their performance is often constrained by radical/electron quenching, incomplete mineralization, and sensitivity to PFAS structure and water chemistry. Biodegradation and enzymatic approaches offer mild transformation pathways but remain limited by slow kinetics, narrow substrate specificity, and uncertain toxicity evolution. Nonradical and thermochemical processes show stronger potential for deep destruction, particularly in concentrated PFAS streams. Overall, electrochemical oxidation, plasma treatment, and thermal/supercritical oxidation appear closer to practical implementation for spent adsorbents, regenerants, industrial concentrates, and other high-strength wastes, whereas many photocatalytic, biological, and microdroplet systems remain laboratory-stage. Future research should prioritize integrated separation–destruction treatment trains and standardized metrics including total organic fluorine removal, fluoride release, final residual PFAS concentrations relative to regulatory thresholds, transformation-product toxicity, energy consumption, and life-cycle impacts.

## 1. Introduction

Perfluoroalkyl substances (PFASs), recognized as persistent and bioaccumulative “forever chemicals” with significant ecotoxicity, are extensively utilized in firefighting foams, nonstick coatings, waterproof materials, and other industrial applications [1,2]. These compounds undergo widespread environmental transport and accumulation and can pose serious health risks—including immune suppression, endocrine disruption, and carcinogenicity—through bioaccumulation [3,4,5]. The environmental recalcitrance of PFASs stems primarily from the high bond energy (~544 kJ·mol^−1^) of the C–F bond and the steric shielding effect of fluorine atoms, which confer strong resistance to conventional degradation pathways and render traditional water treatment processes largely ineffective [6,7,8].

PFASs include compounds with diverse chain lengths, functional groups, and precursor structures, such as legacy perfluoroalkyl carboxylic acids and sulfonic acids, as well as emerging alternatives including GenX and ether-containing PFASs. Long-chain PFASs generally exhibit stronger bioaccumulation and toxicological concerns, whereas short-chain PFASs are more mobile, persistent, and difficult to remove. Therefore, carbon-chain shortening should not be automatically regarded as detoxification [1,2,3,4,5,9,10]. Effective PFAS degradation should ideally achieve extensive C–F bond cleavage, fluoride release, mineralization, and toxicity reduction, rather than merely transforming one PFAS into another fluorinated compound [11,12,13,14,15,16,17,18,19]. Because the degradation pathways and toxicity of many intermediates remain unclear, future studies should combine chemical analysis with toxicity-oriented endpoints, including total organic fluorine removal, transformation-product identification, and residual toxicity assessment.

Current water treatment technologies, including coagulation/flocculation, biological treatment, adsorption, and ion exchange, mainly transfer or concentrate PFASs rather than destroy them, resulting in secondary pollution and costly waste management [9,10,20,21]. Even advanced oxidation processes based on •OH or O_3_ have limited ability to cleave the robust C–F bonds, highlighting the urgent need for efficient destructive treatment technologies [22,23].

Accordingly, this review focuses on four major PFAS degradation pathways in aquatic environments: oxidation-dominated processes, biological transformation coupled with multiprocess interactions, reduction-dominated pathways driven by strong reductants such as hydrated electrons, and nonradical pathways involving direct electron transfer or photogenerated hole oxidation [24,25,26,27]. Beyond summarizing degradation mechanisms, this review further evaluates these strategies using application-oriented criteria, including parent-compound removal, defluorination and mineralization efficiency, validation scale, matrix tolerance, energy and chemical demand, material durability, byproduct control, and secondary-waste management. Because PFAS regulatory limits are generally compound-specific or group-based and differ among jurisdictions, degradation technologies should ultimately be evaluated not only by percentage removal or defluorination, but also by measured final PFAS concentrations, analytical detection limits, fluorine mass balance, and compliance with applicable regulatory thresholds. By linking mechanistic understanding with technology-readiness assessment, this review identifies realistic application windows and future development priorities for sustainable PFAS remediation [11,12,13,17,28,29].

## 2. Core Degradation Mechanisms

**Figure 1:**
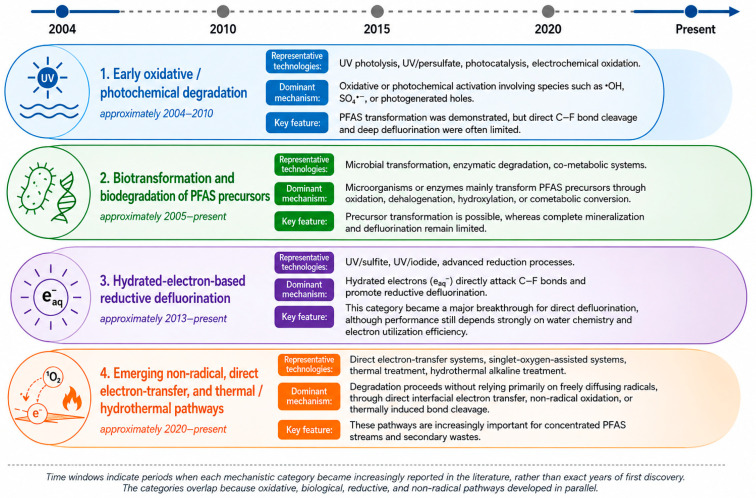
Approximate evolution of major mechanistic concepts and representative technology groups in aqueous PFAS degradation. The time windows indicate periods during which each mechanistic category became increasingly reported in the literature, rather than exact years of first discovery. The categories overlap because oxidative/photochemical, biological, reductive, and non-radical pathways have developed in parallel. Representative technologies and dominant mechanisms are shown to clarify the basis for classifying PFAS degradation technologies.

To clarify the mechanistic basis and temporal rationale of Figure 1, the development of aqueous PFAS degradation research is summarized as an overlapping evolution of major mechanistic concepts rather than a series of strictly separated historical stages. The time windows shown in Figure 1 were assigned according to a chronological mapping of representative studies and the periods during which specific degradation mechanisms became increasingly reported in the literature. Therefore, these windows should not be regarded as exact years of first discovery or as mutually exclusive stages. Instead, oxidative/photochemical, biological, reductive, and non-radical pathways have developed in parallel, with different mechanisms becoming prominent at different periods.

Early studies mainly focused on oxidative and photochemical degradation. Representative work demonstrated the photochemical decomposition of PFOA in water, the persulfate-assisted photochemical oxidation of perfluorocarboxylic acids, and the electrochemical oxidation of PFOS using boron-doped diamond electrodes [30,31,32]. With the development of heterogeneous photocatalysts and photoelectrochemical systems, oxidative/photochemical approaches have expanded to include UV photolysis, UV/persulfate, photocatalysis, electrochemical oxidation, and photoelectrochemical oxidation [32,33]. These technologies generally rely on reactive oxidative species or photogenerated charge carriers, but their performance may be limited by radical scavenging, matrix effects, and incomplete defluorination.

In parallel, biological transformation studies have received increasing attention since the mid-2000s, particularly for polyfluorinated precursors rather than fully fluorinated terminal perfluoroalkyl acids. Studies on fluorotelomer alcohols have shown that microbial processes can transform PFAS precursors into polyfluorinated and perfluorinated products under different environmental or physiological conditions [34,35,36]. These findings highlight the importance of biodegradation for understanding precursor transformation and environmental fate, although complete mineralization and deep defluorination of highly fluorinated terminal products remain challenging.

Since the early 2010s, hydrated-electron-based reductive defluorination has become an important mechanistic direction because hydrated electrons can directly attack electron-deficient C–F bonds. Reductive degradation systems based on iodide photolysis and UV/sulfite chemistry have been shown to promote PFAS defluorination, while subsequent studies further revealed the influence of pH, water chemistry, head group, and chain length on degradation efficiency [37,38,39,40]. These findings support the classification of UV/iodide, UV/sulfite, UV/electrochemical systems, and other advanced reduction processes as a distinct reductive pathway in Figure 1.

More recently, non-radical, direct electron-transfer, and thermal/hydrothermal pathways have attracted growing attention, especially for concentrated PFAS streams and secondary wastes. These approaches involve mechanisms such as interfacial electron transfer, non-radical oxidation, hydrothermal alkaline reactions, and supercritical water oxidation [41,42,43,44]. Compared with direct treatment of dilute environmental waters, they may be more suitable for PFAS concentrates, spent adsorbents, ion-exchange regenerants, AFFF wastes, and other high-strength matrices where destructive treatment is more feasible. From a technology-readiness perspective, electrochemical oxidation, hydrothermal alkaline treatment, and supercritical water oxidation are relatively closer to practical application for concentrated PFAS waste streams, whereas photocatalysis, photoelectrochemical oxidation, biodegradation, and many hydrated-electron-based systems remain mainly at the laboratory or pilot-validation stage.

### 2.1. Oxidation-Driven Pathways

Oxidative mechanisms rely primarily on highly reactive oxidants to attack the C–F bonds or functional groups of PFASs, achieving degradation and mineralization through processes such as bond cleavage, decarboxylation, and chain shortening [45,46]. Various advanced oxidation processes (AOPs) have been employed for this purpose. Table 1 systematically summarizes the key characteristics of these AOPs, including the dominant reactive species, oxidation potential, reaction kinetics, and steady-state concentrations.

A comprehensive analysis of the data in Table 1 indicates that various advanced oxidation processes (AOPs) degrade PFASs primarily through the attack of highly reactive radicals (mainly •OH and SO_4_•^−^) on the carboxyl functional groups, triggering decarboxylation and C–F bond cleavage. The degradation efficiency is governed by both the intrinsic oxidizing power of the reactive species and their effective steady-state concentrations in the system [51,58,66]. Although the oxidation potentials of •OH (~2.8 V vs. NHE) and SO_4_•^−^ (2.5–3.1 V vs. NHE) are thermodynamically sufficient to drive C–F bond cleavage, notable differences in degradation kinetics are observed among different AOPs operating at similar potentials. Electrochemical oxidation using BDD or Ti_4_O_7_ anodes achieves PFOA degradation rate constants in the range of 0.019–0.1838 min^−1^ [11,12,53,55,56,57,58], whereas the rate constants for photocatalytic oxidation typically fall between 0.0037 and 0.1 min^−1^ [47,48,49,50,51]. This contrast clearly demonstrates that the oxidation potential is not the sole determinant of the degradation rate.

The observed differences in degradation kinetics are directly associated with each system’s capacity to generate and maintain steady-state concentrations of reactive species. Plasma oxidation simultaneously produces •OH, O_3_, e_a_q^−^, and reactive nitrogen species, creating a synergistic oxidative-reductive environment that enables PFOA degradation rates of 0.015–0.085 min^−1^, with the effective concentration maintained through gas–liquid interfacial enrichment [60,61,62,63,64,65,66]. Persulfate-based activation techniques primarily generate SO_4_•^−^, achieving PFOA degradation rates of up to 0.1838 min^−1^ while maintaining high steady-state concentrations [11,58,68,69]. Sonolytic oxidation relies on cavitation-induced localized •OH generation and extreme reaction conditions [14,28]; supercritical water oxidation, on the other hand, utilizes •OH under high temperature and pressure to achieve >99.999% PFAS removal [13].

Consequently, the selection of PFAS oxidation technologies requires a systematic evaluation of oxidation capacity, steady-state species concentration, and environmental matrix effects. In actual water bodies, matrix components such as dissolved organic matter (DOM), chloride, and bicarbonate can significantly reduce the effective concentration of reactive species by quenching effects [15,60,66,71]. Future technological development should focus on maintaining high local concentrations of reactive species in complex matrices—for instance, through interfacial enrichment or in situ continuous generation—or on designing more selective reactive species strategies, thereby achieving efficient and deep mineralization of PFAS.

### 2.2. Biodegradation Pathways

Biodegradation has been investigated as a potentially environmentally benign route for PFAS transformation, but its current applicability remains highly limited. Unlike physicochemical methods that rely on exogenous high-energy reactive species, biological processes depend on microbial communities and enzyme systems to mediate PFAS adsorption, transformation, or partial defluorination [16,17,18,19,29,75,76,77,78]. However, these processes are generally slow, substrate-specific, and sensitive to microbial species, redox conditions, cosubstrates, PFAS chain length, functional groups, and experimental conditions. Therefore, the biodegradation performances summarized in Table 2 should be interpreted cautiously, especially when extrapolating laboratory results to complex environmental matrices.

According to dominant biological functions and degradation mechanisms, PFAS biodegradation technologies can be categorized into three main types: biosorption-assisted cometabolism, physicochemical pretreatment-enhanced biotransformation, and direct degradation by specialized defluorinating microorganisms. Biosorption-assisted cometabolism, such as algal–bacterial granular sludge and osmotic microbial fuel cells, relies on extracellular polymeric substances to concentrate PFAS through electrostatic, hydrogen-bonding, and hydrophobic interactions, while microbial metabolic pathways help maintain system activity [29,76,77]. Pretreatment-enhanced biotransformation combines abiotic activation with microbial or enzymatic degradation. For example, photocatalytic–fungal systems can weaken PFAS structures before fungal enzyme-mediated transformation, while microencapsulation can enrich PFOS and protect functional microorganisms or dehalogenases [16,75]. Direct degradation by specialized microorganisms, such as *Acidimicrobium* sp. A6 and *Pseudomonas* species, has also been reported to involve reductive dehalogenase, dehalogenase, or monooxygenase-related pathways that contribute to partial defluorination and chain shortening [17,19,78].

Efficient biological transformation typically begins with interactions between PFAS functional groups and microbial cells, extracellular polymeric substances, or enzyme active sites. Reported pathways may involve attack on characteristic bonds, such as the C–S bond in PFOS or the C–C bond adjacent to the carboxyl group in PFOA, followed by partial defluorination and chain shortening [16,17,18,19]. However, these mechanisms remain incompletely resolved and should not be generalized across different PFAS structures. Overall, PFAS biodegradation is constrained by the high stability of C–F bonds, enzymatic substrate specificity, cosubstrate dependence, long reaction times, and limited effectiveness toward short-chain PFASs. Therefore, biological technologies should be regarded as exploratory or supporting approaches rather than mature stand-alone destructive treatments. Their more realistic role may be as polishing steps, pretreatment-assisted biotransformation systems, or components of integrated physicochemical–biological treatment trains.

### 2.3. Reduction-Driven Pathways

The reductive degradation of per- and polyfluoroalkyl substances (PFASs) relies primarily on highly reductive reactive species, most notably hydrated electrons (e_a_q^−^), to directly attack and cleave strong C–F bonds [40,79]. As summarized in Table 3, the efficacy of this pathway is governed by the reduction potential of key species, electron transfer kinetics, and the energy barrier for defluorination.

The hydrated electron (e_a_q^−^), possessing a highly negative reduction potential of −2.9 V, is the most effective species, with electron transfer rate constants reaching the order of 10^6^–10^9^ M^−1^ s^−1^ [80,81,82,83]. Technologies based on e_a_q^−^ generation, such as UV/VUV/sulfite systems and plasma reduction, exhibit relatively high defluorination kinetics, with pseudo-first-order rate constants up to 0.016–0.37 min^−1^ [87,88,89,102]. Despite these kinetic advantages, the high bond dissociation energy (BDE) of the C–F bond (approximately 106.8–485 kJ/mol or ~5 eV) remains a primary thermodynamic challenge [80,81,85,92]. In contrast, cathodic electrons in electrochemical reduction operate at a less negative potential (−1.8 to −1.4 V vs. SHE), resulting in lower electron transfer rate constants (~10^−4^ s^−1^) and slower defluorination rates [90,91]. Photocatalytic and photoelectrocatalytic systems rely on conduction band electrons (e_cb^−^), whose reduction potential (approximately −0.99 to −0.76 V vs. SCE) further limits the thermodynamic driving force, leading to slower degradation rates (0.2–1.53 h^−1^) [93,95].

Several technologies enhance efficiency through multispecies synergy or interfacial effects. Plasma-cathode electrochemical reduction utilizes energetic gaseous electrons for direct C–F bond attack [92]. The Pd^0^-biofilm synergistic system combines chemical hydrogenation by hydrogen atoms (H•) with the biocatalysis of microbial enzymes [96]. Synergistic processes such as microwave discharge of plasma-Fe^2+^ and UV/zero-valent iron promote the generation and utilization of e_a_q^−^ through the mediation of Fe^2+^ [97,98]. Furthermore, systems such as gas–liquid interface spontaneous reduction systems and solar-driven UV/sulfite systems have the potential for reductive defluorination at specific environmental interfaces or under natural energy driving forces [100].

Collectively, reductive pathways face a dual challenge: sustaining the efficient generation of e_a_q^−^ against quenching by background constituents in real-water matrices while concurrently overcoming the high energy barrier inherent to C–F bond cleavage. Future research should focus on developing more efficient and stable electron donor systems, optimizing interfacial electron transfer processes, and exploring synergistic degradation mechanisms coupled with biological or oxidative processes to achieve efficient and deep defluorination of short-chain and structurally complex PFASs.

### 2.4. Nonradical Pathways

In addition to radical-mediated mechanisms, nonradical pathways constitute another critical route for PFAS degradation (Table 4). These pathways do not rely on diffusible radicals such as •OH or SO_4_•^−^ but instead attack PFAS molecules directly via electron transfer or thermo/chemical activation, particularly by targeting their carboxyl functional groups [103,104].

The adsorption–photocatalytic synergistic technology relies primarily on photogenerated holes (h^+^) at semiconductor surfaces to directly oxidize PFAS. The degradation followed pseudo-first-order kinetics, with rate constants ranging from 0.08 to 0.918 h^−1^ [105,107]. Direct electrochemical oxidation achieves degradation through direct electron transfer at the anode surface, which also results in a broad kinetic range (pseudo-first-order k = 0.0175–0.946 min^−1^) [111,112]. Its efficiency is closely related to the anode material, applied potential, and PFAS structure.

Thermochemical pathways primarily disrupt C–F and C–C bonds directly via thermal energy. Thermal decomposition technology degrades PFAS through pyrolysis at elevated temperatures, with pseudo-first-order rate constants between 0.02 and 0.14 min^−1^ [116,117]. Hydrothermal degradation, which occurs in high-temperature and high-pressure aqueous environments, achieves PFAS transformation via the synergistic effect of OH^−^ nucleophilic attack on the carboxyl group and catalytic decomposition, resulting in a degradation rate constant for PFOA of approximately 0.0204 min^−1^ [124].

In conclusion, while nonradical pathways are distinguished by higher reaction selectivity and robustness against quenching in complex water matrices than their radical counterparts are, their degradation efficiency is frequently constrained by kinetic bottlenecks in interfacial electron transfer or by the magnitude of the applied thermal/chemical energy. These pathways are particularly suitable for treating systems with medium to high PFAS concentrations and low mass transfer limitations or as complementary/synergistic approaches to radical processes. Future research should focus on elucidating the interfacial electron transfer and molecular-level mechanisms and on developing efficient, stable nonradical active interfaces or reaction systems to expand the technological boundaries for PFAS degradation.

## 3. Progress in Degradation Technology and Performance Evaluation

When interpreting the performance data summarized in Table 5, Table 6, Table 7 and Table 8, it should be emphasized that degradation and defluorination percentages indicate destruction potential but do not directly demonstrate regulatory compliance. Compliance assessment requires final measured PFAS concentrations, the relevant target analytes or PFAS groups, analytical detection limits, and the regulatory framework applied in a specific jurisdiction.

### 3.1. Radical Oxidation Technologies

The comparative analysis in Table 5 shows that radical-dominated advanced oxidation technologies (AOTs) can achieve more than 90% removal of PFAS, such as PFOA and PFOS, under optimized laboratory conditions. However, defluorination efficiencies vary widely from 33% to 90%, indicating that parent-compound removal does not necessarily represent complete fluorine mineralization [14]. Most studies remain at the laboratory scale, although electrochemical oxidation using Ti_4_O_7_ anodes and plasma oxidation have shown pilot or transitional-scale potential, maintaining >90% PFAS removal across concentrations from µg/L to mg/L [13]. Nevertheless, their performance toward short-chain and emerging PFASs, long-term stability in complex real-water matrices, energy consumption, and overall cost-effectiveness remain insufficiently demonstrated [11,12].

**Table 5:** Summary and Comparison of PFAS Degradation Performance by Radical-based Oxidation Technologies.

Technology	Target PFAS	Scale	Initial Conc. (mg/L)	Reaction Conditions	Time (h)	Degradation (%)	Defluorination (%)	Refs.
Photocatalytic Oxidation	PFOA	Bench	10	TiO_2_-Pb/rGO, UV 254 nm	24	98	34	[47]
	PFOS	Pilot	0.00013	3D-printed TiO_2_ composite tiles, metal halide lamp	36	90	-	[49]
	PFNA		0.000066		36	89	-	
	PFOA	Bench	0.5	BOHP catalyst, UV 254 nm	2	~100	63	[50]
	PFOA	Bench	0.00414	Fe-zeolite, UV 254 nm	7	100	69	[51]
	PFOS		0.00558			100	51	
	PFOA	Bench	10	chitosan-g-C_3_N_4_, visible light > 420 nm	2	93	37.2	[48]
	PFOS					88	35.2	
Electrochemical Oxidation	PFOA	Bench	0.0206	Ti_4_O_7_ anode, ultrasound-enhanced	6	100	63.5	[48]
	PFOA	Bench	20	BDD anode, 0.1% Na_2_SO_4_, 10 mA/cm^2^	1	68.9	43.4	[11]
	6:2 FTCA					90	~70	
	GenX					65	~55	
	PFBA					50	~40	
	PFHxS	Bench-Pilot	11	BDD electrode, synthetic solution	48	91	-	[12]
	PFOS		15			98	66	
	GenX	Bench	5	BDD anode + Au cathode	2	92	-	[54]
	PFOS	Bench-Pilot	0.00102	Ti_4_O_7_ anode, 5 mA/cm^2^	0.5	90	-	[57]
	PFOA	Pilot	2	Ti_4_O_7_ REM anode, 286 A/m^2^	Short-term	100	-	[53]
	PFOA	Bench	15.6	BDD anode, 60 mA/cm^2^	3	>99	84	[55]
	PFOA	Bench	15.6	BDD anode, 5 mM PS, 40 mA/cm^2^	2	~100	60.4	[58]
	PFOS		19.5			~100	33.1	
	PFOA	Bench-Pilot	0.017	BDD anode, 1 mM Na_2_SO_4_, 0.4–1.0 A	2	100	-	[52]
Plasma Oxidation	PFOS	Bench-Pilot	0.00001	High-voltage DC plasma, air aeration	0.5	>99	-	[61]
	PFOS	Bench	0.000001	Pulsed plasma, needle-plate electrode	0.5	100	70–90	[63]
	PFOA	Bench-Pilot	8.3	Argon discharge, 40 Hz, −30 kV	1	90	-	[65]
	PFOS				0.67	90	-	
	PFBS	Bench	10	Gas–liquid discharge, C_12_TAB, Ar bubbling	0.5	100	-	[59]
	PFOS	Pilot	100	Air GAP discharge, ~300 °C	0.2	>70	~50	[64]
	PFOA	Bench-Pilot	10	Gas–liquid DBD plasma	10	98	-	[62]
	PFOA+PFOS	Bench	0.000579	Non-thermal atmospheric pressure plasma, air carrier gas	0.05	91.2	-	[60]
	PFOS	Pilot	12.59	Continuous-flow SCWO, Ti-lined reactor	2	99.99996	62.6	[13]
	PFOA	Bench	0.1	DBD discharge, 2 mM sulfite, 80 W	0.5	87.9	76.85	[66]
Sonochemical Oxidation	GenX	Bench	1	580 kHz, 400 W/L, pH = 7	1.5	90	90	[14]
	PFOA					85	76	
	PFOS					70	70	
	PFOA	Bench	1	580 kHz, 187.5 W	3	99	90	[28]
	PFOS					91	66	
Persulfate Activation Oxidation	PFOA	Bench	600	60 °C, 30 g/L PS, AC regeneration	24	-	Partial fluoride retention	[69]
	PFOA	Bench	0.63	Fe_3_S_4_ catalyst, 2 mM PMS, pH = 6	1	54	-	[67]
	PFOA	Bench	2	OSBC-800 catalyst, 5 mM PMS, pH = 6.4	0.67	99.5	84	[68]
Enzymatic Catalytic Oxidation	PFOA	Bench	348	HRP enzyme, H_2_O_2_, phosphate buffer pH = 7	6	68	<1	[73]
	PFOA	Pilot Design	0.000005	Laccase enzymatic hydrolysis + BDD electrochemical oxidation	14.2	>99.9	-	[72]
	PFOS					>99.9	-	
Microdroplet Interface Autoxidation	PFOA	Bench	0.0041	Ultrasonic nebulization, room temp., no additives	0.5	72	50	[74]

Future studies should focus on developing durable and low-cost electrode or catalyst materials, improving resistance to matrix interference, and systematically evaluating long-term operational stability and energy-economic performance under realistic water-quality conditions. From a toxicity-control perspective, radical oxidation technologies should be assessed not only by parent PFAS removal, but also by defluorination, mineralization, and the formation of short-chain or partially oxidized fluorinated byproducts, because incomplete oxidation may not necessarily indicate overall risk reduction [11,12,13,14].

### 3.2. Biodegradation Technologies

Biodegradation technologies have attracted interest because they may operate under mild conditions and require lower external energy input than many physicochemical processes. However, PFAS biodegradation remains at an early and highly constrained stage of development. These processes depend on microbial enzyme systems, such as dehalogenases and monooxygenases, and their performance is strongly affected by PFAS structure, microbial community composition, enzyme expression, redox conditions, cosubstrate availability, and reaction time [16,17,18,19,29,75,76,77,78]. As shown in Table 6, biosorption- and cometabolism-based systems, such as algal–bacterial granular sludge and osmotic microbial fuel cells, can achieve 40–60% PFAS removal but require long reaction cycles and show limited direct C–F bond cleavage [29]. In contrast, enhanced biotransformation and specialized microbial degradation pathways exhibit greater defluorination potential; for example, photocatalytic–fungal coupling achieved 90% PFOA degradation and 60% defluorination, while *Acidimicrobium* sp. A6 achieved 60% degradation and 80% defluorination under extended anaerobic cultivation [17,75].

**Table 6:** Efficiency and Mechanisms of PFAS Degradation via Biological Pathways.

Technology	Target PFAS	Scale	Initial Conc. (mg/L)	Reaction Conditions	Time (h)	Degradation (%)	Defluorination (%)	Refs.
Algal–Bacterial Granular Sludge (ABGS) Biosorption & Co-metabolism	PFOA	Bench	1	ABGS, illumination, aeration 1.5 L/min	960	42.6	-	[29]
Photocatalytic Pretreatment + Fungal Co-metabolism	PFOA	Bench	100	Photocatalysis (2 h) + Fungus (48 h)	50	90	60	[75]
Microencapsulated Enzymatic Degradation	PFOS	Bench	2	10% PSf membrane capsule, methanol co-metabolism	1008	80	-	[16]
Osmotic Microbial Fuel Cell	PFOA	Bench	1	OsMFC dual-chamber continuous flow, FO membrane	480	50	Low	[76]
	PFOS					50	Low	
	PFOA	Bench	0.5	Optimized HRT = 10 days	240	60	-	[77]
	PFOS					60	-	
Microbial Enzymatic Degradation	PFOA	Bench-Pilot	0.1	*Acidimicrobium* sp. A6, anaerobic	2400	60	80	[17]
	PFOS		1000	Ensifer adhaerens M1	140	100	40	
	PFOA	Bench-Pilot	1000	Pseudomonas plecoglossicida	96	89.7	13.2	[18]
	PFOS		1.8	*Pseudomonas aeruginosa* HJ4, aerobic	48	67	-	
*Acidimicrobium* sp. A6 Anaerobic Co-metabolic Defluorination	PFDA	Lab Batch	10	A6 enrichment culture, anaerobic	2880	66.3	-	[78]
	PFOA					59.1	-	
	PFOS					39.9	-	
*Pseudomonas* Pure Culture Enzymatic Degradation	PFOA	Lab Batch	0.1	*Pseudomonas aeruginosa*, static culture	96	27.9	-	[19]
	PFOS					47.3	-	

Despite these reported examples, PFAS biodegradation remains less mature than physicochemical degradation technologies. Many studies require reaction times ranging from days to months, and the observed transformation is often limited to specific PFAS structures, microbial strains, enrichment cultures, or optimized experimental conditions [19,29,75,78]. In addition, parent-compound removal does not necessarily indicate complete defluorination or detoxification, because biological transformation may generate short-chain or partially fluorinated intermediates with uncertain persistence and toxicity [16,17,18,78]. Therefore, biodegradation is unlikely to serve as a near-term stand-alone destructive technology for PFAS remediation. Future work should focus on verifying transformation pathways, improving reaction rates and substrate spectra, quantifying fluoride release and toxicity evolution, and evaluating long-term stability in realistic water matrices.

### 3.3. Reductive Degradation Technologies

Reductive pathways based on hydrated electrons (e_a_q^−^) have shown strong PFAS defluorination potential under laboratory conditions. As summarized in Table 7, sulfite photoreduction and plasma reduction can achieve >90% degradation and 58.9–98% defluorination for various long-chain PFASs [59,65,80,82,83,85,87]. Synergistic systems, such as DBD/sulfite–ultrafiltration coupling, can further increase PFOA defluorination to 98%, indicating the performance-enhancing effect of multiprocess integration [101].

**Table 7:** Performance Evaluation of Reductive Technologies for PFAS Treatment.

Technology	Target PFAS	Scale	Initial Conc. (mg/L)	Reaction Conditions	Time (h)	Degradation (%)	Defluorination (%)	Refs.
Sulfite Photoreduction	PFOS	Bench	10	UV/VUV, 10 mM SO_3_^2−^, 22 °C	6	64	58.9	[80]
	PFHxS					52.6	36.8	
	6:2 FTSA					37.8	28.4	
	PFBS			UV/VUV, 10 mM SO_3_^2−^, 10 mM KI, 85 °C		38	18	
	PFOA	Bench	0.1	pH = 12, 10 mM SO_3_^2−^	6	100	66.2	[81]
	PFBA					99.6	47.8	
	GenX					70	42.3	
	PFHpA	Bench	5.1	VUV/UV, 10 mM SO_3_^2−^, subsequent oxidation	8	83.5	74	[83]
	PFHxS		5.2		8	74	73.4	
	6:2 FTS		5.4		12	98.9	86.9	
	PFOA	Bench	5	UV, 1.5 mg/L BrO_3_^−^	4	96	96	[82]
	PFOS					90	90	
	PFOA	Bench	36 μM	UV, SO_3_^2−^	12	100	73.8	[84]
	PFOS		32 μM		0.5	98	55	
	GenX		0.16 mM		6	100	90	
	PFOS		1.2 mM	Visible light 405 nm, CdS NCs	8	100	100	
	PFOA	Bench	36 μM	VUV, 20 μM Fe^3+^	4	51.2	51.2	[85]
	PFOS		32 μM	UV, SO_3_^2−^	0.5	98	70	
	PFOA	Bench	1	pH = 10, UV irradiation	1	100	98	[71]
Plasma Reduction	F-53B	Bench	0.5	Discharge power 94 W, gas–liquid interface	0.33	100	~45	[89]
	PFOS		10		1.33	89.5	48.3	
	PFOA	Bench	0.2	N_2_ plasma, 60 min	1	45	-	[126]
	PFDA		0.2	N_2_ plasma, 60 min	1	60	-	
	PFDA	Bench	0.0062	Bipolar discharge, Hyamine 1622, air	1.25	>97	82	[86]
	PFNA		0.0118			>97	82	
	PFOS		0.0087			>97	82	
	PFOA		0.0163			>97	82	
	PFHpA		0.0139			>97	82	
	PFBS		0.0191			95	82	
	PFBA		0.0103			53	82	
	PFOS	Pilot	0.1365	Fine bubble, 2 J/pulse, Ar 1 L/min	2	>99.5	-	[102]
	PFOS	Bench	20	20 kV, 50 ns, 1 kHz, Ar	1	100	-	[127]
	PFOS	Bench	10	30 W, Bi_2_O_3_ catalyst, 25 °C	0.5	96.6	68.6	[87]
	PFOA	Bench	41.4	Ar bubbling, 4 W input, room temp.	0.5	98.9	64.4	[59]
	PFOS		5		0.17	>99	-	
	PFBS	Bench	10	Gas–liquid discharge, 0.2 mM C_12_TAB, 10 °C, Ar	0.5	100	-	[90]
	PFOA	Pilot	8.3	Ar 4 L/min, 40 Hz, −30 kV, room temp.	1	90	-	[65]
	PFOS				1	90	-	
Electrochemical Reduction	PFMeUPA	Bench	49.5	10 V, 70 °C, 0.1 M Na_2_SO_4_	5	100	~90	[90]
	PFOS		50	15 V, 85 °C, 0.1 M Na_2_SO_4_, 250 μM B12	4	7.9	7.9	
	GenX	Bench	5	BDD anode + Au cathode, 0.1 M Na_2_SO_4_+NaCl	2	92	-	[54]
	PFOA	Bench	413	pH = 9.5, 0.5 M KHCO_3_, Au electrode, −1.80 V	14	100	~35	[91]
Plasma-Cathode Electrochemical Reduction	PFOA	Bench	6.2	Plasma cathode, 2 mA, Ar purge	2	100	~60	[92]
Photocatalytic/ Photoelectrocatalytic Reduction	PFOS	Bench	40	365 nm LED, TEOA	24	99	97	[93]
	PFOA	Bench	10	UV, pH = 6, ZnS-40% [N]	3.5	>90	>80	[94]
	PFOS				3	>90	85	
	PFHxA					>90	70	
	6:3-FTCA					>90	60	
	HFPO-TA					90	30	
	PFBA					90	40	
	PFOA	Bench	0.5	Simulated sunlight, PMS activation, pH 3–9	0.67	93.6	56.7	[95]
Pd^0^-Biofilm Synergistic Reduction	PFOA	Bench	0.0041	H_2_ MBfR, pH = 7.2, co-existing nitrate	24	85	81	[96]
Microwave Discharge Plasma-Fe^2+^ Synergistic Reduction	PFOA	Bench	40	150 W, pH = 3.8, 10 mg/L Fe^2+^	1.67	98.6	82.9	[97]
UV/Zero-Valent Iron Photocatalytic Reduction	PFNA	Bench	0.0005	UV 254 nm, 100 mg/L Fe^0^, pH = 3	2	90	-	[98]
	PFOS					88	-	
	PFOA					46	-	
UV/DIHA Heterogeneous Photoreduction	PFOA	Bench	10	UV 254 nm, DIHA nanospheres, pH 4–10	12	100	79	[99]
	PFOS					100	79	
	6:3-FTCA					100	66	
	HFPO-DA					100	45	
	PFBS					51	51	
	TFA					9	9	
Gas–Liquid Interface Spontaneous Reduction	PFOA	Bench	0.02	Microdroplet, pH = 9.8, 0.1 mM NaBr, air	Short	36.4	36.4	[100]
Solar-Driven UV/Sulfite Reduction	PFOA	Pilot	1	pH = 10, solar UV irradiation	24	100	89	[15]
DBD/Sulfite–Ultrafiltration Coupled Process	PFOA	Bench/Pilot	0.1	Sulfite 2 mM, discharge power 80 W, Ar 2 L/min	1.5	87.9	98	[101]

Despite this potential, practical application remains constrained by substrate specificity, limited effectiveness toward short-chain and emerging PFASs, and the fact that most high-efficiency results are still obtained under well-controlled bench-scale conditions. Scale-up also faces challenges such as e_a_q^−^ quenching in complex water matrices, high energy consumption, and incomplete degradation products. Therefore, future research should focus on developing selective and interference-resistant catalysts or electrode materials, optimizing reactor design to improve electron utilization and energy efficiency, and verifying long-term adaptability in real-water matrices. In addition, comprehensive solutions should be developed to integrate pollutant removal with fluorine resource recovery. Accordingly, reductive technologies should be coupled with transformation-product identification and toxicity evaluation to confirm whether high defluorination efficiency is accompanied by genuine risk reduction rather than the accumulation of partially defluorinated intermediates [80,81,82,83,84,85,97,98,99,100,101].

### 3.4. Nonradical Pathways Technologies

Nonradical pathways degrade PFAS through direct electron transfer, pyrolysis, or hydrothermal decomposition without relying on diffusible free radicals. As summarized in Table 8, adsorption–photocatalytic synergy and electrochemical direct oxidation can achieve 79.2–100% degradation of PFAS such as PFOA through photogenerated holes or anodic electron transfer, with some systems reaching the pilot scale [105,107,109,111,115]. Thermochemical pathways, including thermal decomposition and hydrothermal degradation, directly break C–F bonds under high-energy conditions. At 600–1000 °C for thermal decomposition or 240–325 °C for hydrothermal degradation, these processes can achieve >99% PFAS removal and 85–97% defluorination, indicating strong mineralization potential [116,117,119,124].

**Table 8:** Summary of PFAS Degradation Efficiency through Non-radical Pathways.

Technology	Target PFAS	Scale	Initial Conc. (mg/L)	Reaction Conditions	Time (h)	Degradation (%)	Defluorination (%)	Refs.
Adsorption-Photocatalytic Synergy	PFOA	Bench	100	254 nm UV, In/TNTs@AC	4	>99	60	[105]
	PFOA	Bench	100	254 nmUV, Fe/TNTs@AC	4	91.4	62	[106]
	PFOA	Bench	0.01	Visible light, ZIF67@C_3_N_4_	6	79.2	-	[109]
	PFOA	Bench	0.2	254 nm UV, BiOHP/CS	4	91	32.5	[110]
	PFOA	Pilot	66	254 nm UV, BOHP	1	>99	60	[108]
	PFOA	Bench	1	254 nm UV, Fe/g-C	6	89.7	42.2	[107]
Electrochemical Direct Oxidation	PFOA	Bench	10	BDD anode, Na_2_SO_4_	4	99.5	50	[114]
	PFOA	Pilot	100	BDD anode, pH = 2.5	6	99.5	60	[115]
	PFOA	Bench	0.14	BDD anode, 100 mA/cm^2^	4	100	-	[112]
	GenX	Bench	86.8	BDD anode, Gr-Ni-foam cathode	3	100	89	[113]
	PFOS	Bench	11	Ti_4_O_7_ anode, UV irradiation	6	100	-	[111]
Thermal Decomposition	Total PFAS	Bench	0.00041	600 °C, N_2_ atmosphere	1	99.89	85	[117]
	PFOA	Bench	5	250 °C, with GAC addition	1	>99	-	[119]
	PFOA	Bench	-	700 °C, GAC supported	0.5	>99.9	92.4	[122]
	PFOS	Bench	0.265	1000 °C, H_2_O(g)	0.014	100	97	[120]
	PFOA	Bench	-	700 °C, GAC supported	0.5	>99.9	92.4	[118]
	HFPO-DA	Bench	-	200 °C, GAC supported	1	90	-	[116]
	Total PFAS	Bench	-	400 °C pyrolysis	-	>99	~100	[121]
Hydrothermal Degradation	PFOA	Bench	200	240 °C, 1M NaOH	0.5	100	97	[125]
	PFBS	Bench	80	325 °C, 2M NaOH	1.5	99.4	-	[123]
	PFOA	Bench	0.0002	270 °C, 30 min	0.5	>99	-	[124]

However, their practical application is limited by high energy and material inputs, stringent equipment requirements, and greater suitability for high-concentration point sources rather than dilute environmental waters. Future studies should focus on developing low-temperature catalytic systems, improving the selectivity, stability, and lifetime of photoelectrochemical/electrochemical interfaces, and integrating destructive technologies with low-energy enrichment methods such as adsorption to build more economically viable treatment trains. For nonradical and thermochemical pathways, toxicity reduction is more likely when deep mineralization or near-complete fluorine release is achieved; however, for interfacial or catalytic systems with partial conversion, transformation-product toxicity should still be systematically assessed [105,106,107,108,109,110,111,112,113,114,115,116,117,118,119,120,121,122,123,124,125].

To further relate the degradation efficiencies in Table 8 to regulatory relevance, it should be noted that PFAS limits vary substantially among jurisdictions and are generally defined at the ng/L level for drinking water. For example, representative values include the U.S. EPA maximum contaminant levels of 4 ng/L for PFOA and PFOS and 10 ng/L for PFHxS, PFNA, and HFPO-DA [128], the EU Drinking Water Directive values of 0.1 µg/L for the Sum of PFAS and 0.5 µg/L for PFAS Total [129], the Health Canada objective of 30 ng/L for the sum of 25 specified PFAS [130], and the Australian guideline values of 200 ng/L for PFOA, 8 ng/L for PFOS, 30 ng/L for PFHxS, and 1000 ng/L for PFBS [131]. Therefore, regulatory compliance cannot be determined from degradation or defluorination percentages alone, but should be assessed using measured final concentrations, analytical detection limits, and the specific PFAS compounds included in each regulatory framework.

When the initial concentration and degradation efficiency reported in Table 8 are used to estimate the residual parent-PFAS concentration as residual = C0 × (1 − degradation/100), most high-concentration bench-scale studies remain difficult to interpret from a compliance perspective. For instance, even >99% degradation of PFOA from an initial concentration of 100 mg/L could still correspond to a residual concentration below 1 mg/L, which is far above ng/L-level drinking-water criteria. Similarly, entries reported as “100%” degradation cannot be considered compliant unless the final concentration and method detection limit are provided. In contrast, the low-initial-concentration thermal decomposition entry for total PFAS (0.00041 mg/L, 99.89% degradation) gives an estimated residual concentration of approximately 0.45 ng/L, and the hydrothermal degradation entry for PFOA (0.0002 mg/L, >99% degradation) gives an estimated residual concentration below 2 ng/L. These values are below the representative regulatory limits listed above, subject to confirmation by measured final concentrations and the applicable regulatory target analytes. Thus, Table 8 demonstrates the destruction potential of nonradical and thermochemical processes, but regulatory compliance should be claimed only when final PFAS concentrations, fluorine mass balance, and transformation-product toxicity are adequately reported.

### 3.5. Critical Synthesis: Technology Readiness and Realistic Application Windows

Although the technologies summarized above have demonstrated potential for PFAS degradation, their practical values differ substantially. High parent-compound removal under optimized laboratory conditions does not necessarily indicate engineering readiness, because incomplete defluorination, high energy input, poor matrix tolerance, limited material durability, and the formation of short-chain or partially fluorinated byproducts may restrict field application [11,12,13,14]. Therefore, practical applicability should be assessed using multiple criteria, including defluorination and mineralization efficiency, validation scale, operational stability in real-water matrices, energy and chemical consumption, byproduct control, and secondary-waste management [9,10,13,17,29]. Based on these criteria, Table 9 classifies the reviewed technologies according to their approximate technology readiness, energy demand, relative cost, realistic application windows, and major barriers.

Electrochemical oxidation, plasma-based treatment, and thermal or supercritical water oxidation appear relatively closer to practical implementation than most other destructive technologies. Electrochemical oxidation and plasma processes have shown high PFAS removal at bench or pilot-related scales and can be integrated into modular treatment units [11,12,13,52,53,54,55,56,57,58,59,60,61,62,63,64,65,66,111,112,113,114,115]. However, their wider application is still limited by electrode cost, energy consumption, mass-transfer efficiency, and long-term stability in complex matrices [11,12,13]. Thermal and supercritical water oxidation can achieve deep destruction and are more suitable for concentrated PFAS waste streams, spent adsorbents, ion-exchange regenerants, or industrial concentrates, but they are less practical for direct treatment of large volumes of dilute contaminated water because of their high energy demand and demanding reactor conditions [13,116,117,118,119,120,121,122,123,124,125].

Other technologies, including UV/VUV/sulfite photoreduction, sonochemical degradation, persulfate activation, and selected nonradical catalytic or electrochemical systems, represent transitional approaches with strong mechanistic promise but insufficient field validation [14,15,28,69,70,71,80,81,82,83,84,85,93,94,95]. Their practical performance remains sensitive to pH, dissolved organic matter, bicarbonate, chloride, coexisting ions, and light or energy penetration [15,80,81,112,113]. Therefore, future work should move beyond demonstrating PFAS disappearance in model solutions and instead verify stable defluorination, acceptable energy cost, and controllable byproduct formation during continuous treatment of real waters.

Biodegradation, enzymatic degradation, microdroplet interfacial reactions, and many photocatalytic or photoelectrocatalytic systems remain mainly at the laboratory or proof-of-concept stage. Although these technologies are attractive because of their potential selectivity, mild reaction conditions, or lower chemical input [16,17,18,19,29,47,48,49,50,51,72,73,74,75,76,77,78,93,94,95,105,106,107,108,109,110], their slow kinetics, narrow substrate specificity, long reaction times, dependence on idealized conditions, and limited evidence for complete mineralization make them less suitable as stand-alone near-term remediation options [17,19,29,72,73]. Their more realistic role may be as polishing steps, pretreatment-assisted systems, or components of hybrid treatment trains.

Adsorption and ion exchange should be considered as supporting separation technologies because PFAS contamination in raw water, groundwater, and drinking-water sources often occurs at low concentrations. These mature technologies can capture and concentrate PFASs, particularly long-chain compounds, from large volumes of dilute water [9,10]. However, they mainly transfer PFASs to adsorbents or regenerants rather than cleaving C–F bonds. Thus, their practical role is better defined as front-end separation and enrichment, followed by downstream destructive treatment of spent media, regenerants, or concentrates.

Overall, no single degradation technology can currently serve as a universal solution for PFAS-contaminated water. For dilute environmental waters, a realistic strategy is likely to involve adsorption or ion exchange for separation and enrichment, followed by destructive treatment of concentrated PFAS streams [9,10]. For high-strength wastes, spent adsorbents, ion-exchange regenerants, and industrial concentrates, energy-intensive destructive processes such as electrochemical oxidation, plasma treatment, or thermal/supercritical oxidation may be more appropriate [11,12,13,52,53,54,55,56,57,58,59,61,62,63,64,65,66,116,117,118,119,120,121,122,123,124,125]. Future studies should therefore shift from isolated degradation efficiencies toward integrated treatment trains evaluated by standardized metrics, including total organic fluorine removal, fluoride recovery, toxicity evolution, energy consumption per unit PFAS destroyed, material lifetime, and life-cycle environmental impacts.

## 4. Critical Factors and Challenges in the Translation of Technology from the Laboratory to Practice

Based on the TRL-based application-oriented assessment in Table 9, the key challenge in PFAS remediation has shifted from demonstrating degradation mechanisms under controlled laboratory conditions to developing robust, economical, and scalable treatment systems. Degradation efficiency alone cannot determine practical applicability, because technologies with high PFAS removal may still be limited by high energy demand, costly electrodes or reactors, strict matrix requirements, insufficient scale-up validation, incomplete defluorination, byproduct formation, and secondary-waste management. Therefore, practical evaluation should consider technology readiness, energy demand, cost, long-term stability, matrix tolerance, byproduct control, and waste management.

Treatment scenarios, especially initial PFAS concentration and water-matrix composition, are also critical. The studies summarized in Table 5, Table 6, Table 7 and Table 8 cover a wide concentration range, from environmentally relevant trace levels to highly concentrated residual streams. Many laboratory studies use μg/L–mg/L PFAS concentrations to facilitate kinetic analysis, product identification, and fluoride mass-balance evaluation, whereas raw water, groundwater, and wastewater effluents often contain lower PFAS levels, and industrial wastewater, spent adsorbents, ion-exchange regenerants, foam fractions, and other residuals may contain much higher loads [10,11,12,13,14,17,29,57,125]. Thus, laboratory degradation efficiencies should be interpreted together with initial concentration, mass-transfer limitations, and matrix complexity. In general, dilute waters are more suitable for separation/enrichment-based front-end treatment, while destructive technologies are more realistic for concentrated residuals, regenerants, spent adsorbents, foam fractions, and industrial waste streams.

**Figure 2:**
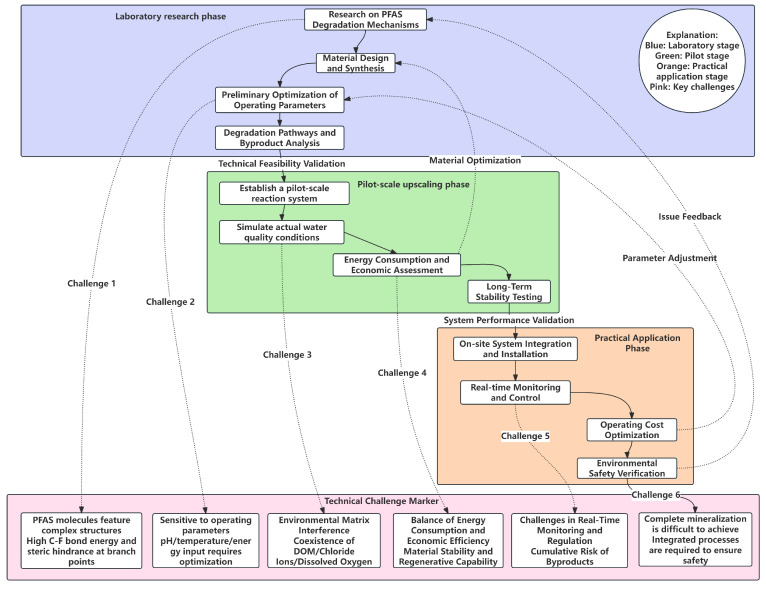
Decision Pathway from Laboratory to Field-Scale Application.

**Figure 3:**
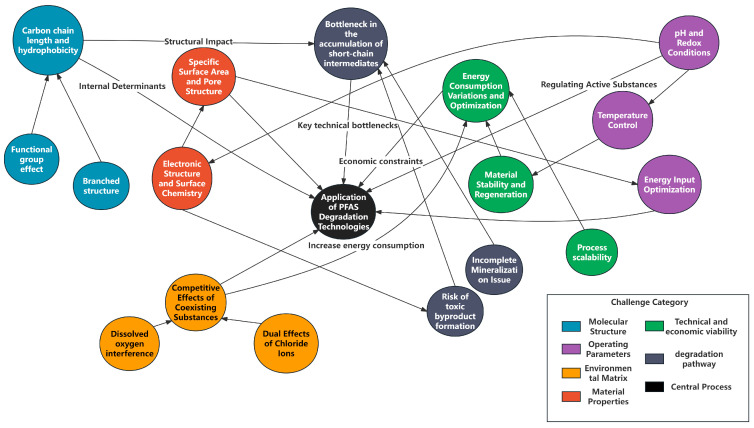
Network Diagram of Integrated Challenges Facing PFAS Degradation Technology Application.

The major PFAS discharge sources include firefighting foams, fluorochemical manufacturing, nonstick and waterproof material production, landfill leachate, wastewater treatment plant effluents, and secondary concentrated wastes from adsorption or ion exchange [1,2,9,10,20,21]. Depending on the source, PFAS-containing waters may contain dissolved organic matter, suspended solids, chloride, bicarbonate, sulfate, nitrate, dissolved oxygen, coexisting organic contaminants, surfactants, salts, and metal ions. These components can inhibit degradation by scavenging radicals, quenching hydrated electrons, competing for active sites, altering interfacial adsorption, or promoting undesired byproducts [12,15,80,81,112,113]. As illustrated in Figure 2 and Figure 3, PFAS degradation is jointly controlled by contaminant structure, reaction conditions, environmental matrix, material properties, technoeconomic viability, and byproduct risks. Accordingly, this section discusses the key factors governing the transition of PFAS degradation technologies from proof-of-concept studies to realistic field implementation.

### 4.1. Influence of the Pollutant Molecular Structure

The molecular structure of PFAS is the intrinsic determinant of its degradation behavior, occupying a central position in the integrated challenge network shown in Figure 3. The carbon chain length directly influences the hydrophobicity and interfacial adsorption propensity. Typically, long-chain PFASs (e.g., PFOA and PFOS) exhibit shorter degradation half-lives, whereas short-chain analogs (e.g., PFBA and TFA) are more difficult to remove effectively because of their higher water solubility and mobility [80,112]. The chemical nature of functional groups (e.g., sulfonate vs. carboxylate) further influences the interaction mechanisms with reactive species or catalytic material surfaces by modulating the molecular charge distribution and spatial configurations [81,113]. Additionally, the steric hindrance introduced by branched structures generally slows degradation kinetics, while relatively weaker chemical bonds within the molecule (e.g., ether bonds, C–H bonds) often become key sites for initiating degradation chain reactions, governing the overall reaction pathways and rates [81,113].

### 4.2. Regulatory Role of Operational Parameters in Process Performance

The operational parameters of a degradation system are key external variables for controlling the reaction kinetics and selectivity. Their optimization pathways and strategies run through the decision-making process from the laboratory to scale-up applications, as outlined in Figure 2. The solution pH not only affects the ionic state of PFAS but also profoundly affects the stability and generation efficiency of reactive species (e.g., hydrated electrons, e_a_q^−^; holes, h^+^) [80,81]. In thermochemical processes, temperature directly influences the activation energy, with PFASs of different structures having distinct pyrolysis thresholds [119,120]. For technologies driven by external fields such as electrochemistry and plasma, the current density and discharge power require fine-tuning to maximize the yield of active species while suppressing undesired side reactions triggered by energy overload [113].

### 4.3. Constraining Effects of the Environmental Matrix on Process Efficiency

The complex composition of real PFAS-containing waters is a major bottleneck for applying degradation technologies. As shown in Figure 3, this issue corresponds to “environmental matrix interference” and “competition from coexisting substances” in the integrated challenge network. Different discharge sources, such as industrial wastewater, firefighting-foam-impacted water, landfill leachate, wastewater treatment plant effluents, and concentrated residual streams, may contain dissolved organic matter (DOM), chloride, bicarbonate, sulfate, nitrate, suspended solids, dissolved oxygen, surfactants, metal ions, and other organic contaminants. These components can inhibit PFAS degradation by scavenging reactive species, quenching hydrated electrons, competing for active sites, or altering interfacial mass transfer [12,15,80,81,112,113].

Among these matrix constituents, DOM can consume oxidizing radicals, block photocatalyst or electrode surfaces, reduce light utilization, and compete with PFASs for adsorption at reactive interfaces [15,66,71]. Inorganic ions can also affect degradation efficiency and product profiles. Chloride may form reactive chlorine species but can also generate undesired halogenated or oxychlorine byproducts under electrochemical or advanced oxidation conditions [12]. Bicarbonate and carbonate can convert highly reactive radicals into less reactive carbonate radicals, while dissolved oxygen and nitrate can compete with PFASs for hydrated electrons in reduction-dominated systems, thereby lowering electron utilization and defluorination efficiency [15,80,81,82,83,84,85].

Solution pH, salinity, and coexisting micropollutants further influence PFAS degradation by changing PFAS ionization, catalyst or electrode surface charge, reactive-species generation, conductivity, interfacial mass transfer, and active-site competition [12,80,81,112,113]. Therefore, high removal efficiencies obtained in simplified matrices should be interpreted cautiously. Future studies should validate PFAS degradation technologies in representative real waters or simulated matrices containing relevant DOM, inorganic ions, salinity, pH conditions, and coexisting micropollutants.

### 4.4. Pivotal Role of Material Properties in Determining Performance and Functionality

In heterogeneous catalytic and electrochemical systems, the physicochemical properties of materials directly determine their degradation efficacy. The screening and optimization in this dimension are closely linked to the “Material Evaluation and Process Compatibility” step in Figure 2. Microstructural features (e.g., specific surface area and pore size distribution) govern the adsorption capacity and mass transfer of PFASs [106,109]. The electronic structure and surface chemical state of materials (e.g., doping type, oxygen vacancy density, cycling efficiency of surface redox pairs) fundamentally regulate charge carrier separation, interfacial electron transfer, and the generation kinetics of key reactive species, thereby determining the activity and pathway selectivity of catalytic reactions [75,96].

### 4.5. Technoeconomic Assessment and Optimization for Sustainable Implementation

Promoting the engineering application of degradation technologies necessitates systematic technoeconomic analysis, a process directly corresponding to the node “Assessment of Economic Feasibility and Sustainability” in Figure 2. The specific energy consumption varies significantly among different technologies, ranging from approximately 0.16 kWh/m^3^ for highly efficient coupled systems to over 1600 kWh/m^3^ for some physical separation processes [120]. Energy reduction strategies include developing low-energy consumption combined processes (e.g., plasma–biological coupling) and optimizing energy input modes and reactor design [61]. Moreover, the long-term stability and regenerability of materials are critical for determining operational costs. Materials with good cyclic performance and simple regeneration methods hold greater potential for large-scale applications.

### 4.6. Control of Degradation Pathways and Associated Byproduct Risks

Ensuring environmental safety requires systematic monitoring of PFAS transformation pathways and byproduct profiles, corresponding to the “Byproduct Generation and Risk Control” node in Figure 3. PFAS degradation may generate short-chain intermediates with high solubility and stability, such as PFHxA and PFBA, which hinder complete mineralization [81,82]. Other potential byproducts, including volatile fluorinated organics from pyrolysis, perchlorate/bromate from electrochemical processes, and short-chain fluoroethers from biotransformation, should also be carefully controlled [75,119].

The disappearance of parent PFAS should not be regarded as complete detoxification when fluorinated transformation products remain. Partial degradation may produce short-chain perfluoroalkyl acids, partially defluorinated intermediates, fluorotelomer-derived products, or ether-containing transformation products, which may retain high persistence, mobility, and uncertain toxicity [3,4,5,75,81,82]. Therefore, degradation performance should be evaluated together with transformation-product identification, fluorine mass balance, fluoride release, total organic fluorine reduction, and toxicity evolution to determine whether a treatment process truly reduces environmental and health risks rather than merely converting one persistent fluorinated compound into another.

Pathway control requires both mechanistic regulation and risk-oriented evaluation. Oxidative and nonradical systems should be optimized to enhance deep defluorination and mineralization while minimizing short-chain fluorinated products or halogenated byproducts. For reductive and biological systems, partially defluorinated intermediates and precursor-derived products require particular attention [75,81,82]. Future studies should report not only parent-compound removal and defluorination efficiency, but also the identities, persistence, and toxicity evolution of transformation products, especially short-chain and emerging PFAS intermediates.

## 5. Conclusions and Future Perspectives

The effective degradation and deep mineralization of per- and polyfluoroalkyl substances (PFASs) depend on overcoming the exceptional stability of C–F bonds. This review summarizes four major degradation pathways: oxidation, biodegradation, reduction, and nonradical processes. Oxidation technologies enable rapid degradation but are vulnerable to matrix-induced radical quenching and material costs. Biodegradation offers a potentially mild route for PFAS transformation, but its current applicability is limited by slow kinetics, substrate specificity, microbial and redox dependence, and uncertain intermediate toxicity, making it more suitable as a supporting or polishing step than as a stand-alone solution. Reductive technologies based on hydrated electrons show strong defluorination potential, especially for long-chain PFASs, but remain sensitive to PFAS structure and water-matrix interference. Nonradical and thermochemical pathways can achieve deep destruction under suitable conditions, particularly for concentrated PFAS wastes.

A key conclusion is that PFAS degradation technologies should not be evaluated solely by parent-compound removal under optimized laboratory conditions. Practical assessment should also consider defluorination and mineralization efficiency, technology readiness, energy demand, cost, matrix tolerance, material durability, byproduct control, secondary-waste management, and long-term operation. Electrochemical oxidation, plasma treatment, and thermal/supercritical oxidation are relatively closer to practical implementation for concentrated PFAS streams, spent adsorbents, ion-exchange regenerants, foam fractions, and industrial concentrates. In contrast, UV/VUV/sulfite photoreduction, sonochemical degradation, persulfate activation, and selected nonradical catalytic systems require further continuous-flow and real-water validation, whereas biodegradation, enzymatic degradation, microdroplet reactions, and many photocatalytic systems remain mainly at the laboratory or proof-of-concept stage.

PFAS treatment strategies should be matched to contamination scenarios. For low-concentration raw water, groundwater, and drinking-water sources, adsorption and ion exchange are more realistic front-end separation and enrichment technologies, but downstream destructive treatment of spent media, regenerants, or concentrates is still required. For high-strength PFAS wastes, direct destructive technologies such as electrochemical oxidation, plasma treatment, hydrothermal treatment, and supercritical water oxidation may be more appropriate.

From a toxicity- and compliance-oriented perspective, PFAS remediation should not be judged only by parent-compound disappearance, carbon-chain shortening, or percentage removal. Future studies should include final residual PFAS concentrations, analytical detection limits, fluorine mass balance, fluoride recovery, total organic fluorine reduction, transformation-product identification and toxicity, and comparison with jurisdiction-specific regulatory thresholds to better confirm practical compliance and genuine environmental and health risk reduction.

Future research should advance synergistically across the following fronts:

(1) Materials and reactor innovation. Durable, low-cost, and selective electrodes, catalysts, membranes, and functional materials should be developed to improve PFAS degradation efficiency, electron or radical utilization, matrix resistance, and long-term stability. Reactor design should also be optimized to enhance mass transfer, energy efficiency, scalability, and continuous operation.
(2) Scenario-oriented technology integration. Treatment systems should be designed according to PFAS concentration, matrix composition, and waste-stream characteristics. For dilute waters, an “enrichment–destruction–polishing” strategy is more realistic, whereas concentrated PFAS residuals may be more suitable for direct destructive treatment. Coupling separation with electrochemical, plasma, thermal, reductive, or biological polishing processes may improve both efficiency and economic feasibility.
(3) Real-water and engineering-scale validation. Future studies should move beyond simplified laboratory matrices and evaluate long-term performance in representative real waters containing dissolved organic matter, inorganic ions, salinity, surfactants, suspended solids, and coexisting micropollutants. Pilot-scale and continuous-flow validation, together with standardized metrics such as energy consumption per unit PFAS destroyed, fluoride recovery, material lifetime, treatment cost, and life-cycle impacts, is needed to bridge laboratory performance and field application.
(4) Pathway control and risk management. Greater attention should be paid to degradation pathways, intermediate evolution, fluorine mass balance, and byproduct toxicity. Reaction conditions should be regulated to promote deep defluorination and mineralization while minimizing persistent short-chain or partially fluorinated products. Comprehensive monitoring is needed to ensure genuine risk reduction.

Overall, no single technology can currently serve as a universal solution for PFAS-contaminated water. Future progress will depend on matching mechanisms with realistic application scenarios, integrating separation and destruction processes, validating performance under complex real-water conditions, and evaluating treatment outcomes using both chemical and toxicity-oriented indicators.

## Figures and Tables

**Table 1:** Mechanisms and Kinetic Characteristics of Active Species Formation in PFAS Advanced Oxidation Processes.

Technology	Key Reactive Species	Oxidation Potential	Rate Constant with Functional Group	Steady-State Concentration of Reactive Species	Refs.
Photocatalytic Oxidation	h^+^, •OH, •O_2_^−^, ^1^O_2_	2.7–3.2 V vs. NHE (VB hole)	PFOA: 0.0037–0.1 min^−1^ (pseudo-first-order)	Medium-High, effective concentration maintained by suppressed carrier recombination	[47,48,49,50,51]
Electrochemical Oxidation	•OH, SO_4_•^−^, direct electron transfer	>2.0 V vs. SHE (anodic potential)	PFOA: 0.019–0.1838 min^−1^ (pseudo-first-order)	High, stable steady-state concentration from continuous anodic generation	[11,12,52,53,54,55,56,57,58]
Plasma Oxidation	•OH, O_3_, e_a_q^−^, RNS	•OH 2.8 V, O_3_ 2.07 V vs. NHE	PFOA: 0.015–0.085 min^−1^ (pseudo-first-order)	Medium-High, sustained generation and interfacial enrichment via discharge	[13,59,60,61,62,63,64,65,66]
Sonolytic Oxidation	•OH, pyrolytic radicals	Equivalent > 3 V (pyrolytic energy)	GenX: 0.0501 min^−1^, PFOA: 0.0444 min^−1^	Medium, localized high concentration in cavitation bubbles	[14,28]
Persulfate Activation	SO_4_•^−^, •OH, ^1^O_2_	SO_4_•^−^ 2.5–3.1 V vs. NHE	PFOA: 0.1838 min^−1^ (electro-activated)	High, continuous generation in activated systems	[11,58,67,68,69]
Ozonation	O_3_, •OH	O_3_ 2.07 V vs. NHE	Relatively low, often for precursor transformation	Quenched by dissolved organic matter (DOM)	[15,70,71]
Enzymatic Oxidation	•OH, enzyme active center	•OH 2.8 V vs. NHE	PFOA: ~0.003 min^−1^ (HRP)	Low, dependent on co-substrates	[72,73]
Supercritical Water Oxidation	•OH (high T&P)	Energy provided by high T&P	DRE > 99.999% (Destruction and Removal Efficiency)	High, sustained generation in supercritical state	[13]
Microdroplet Interfacial Autoxidation	•OH, e^−^ (interfacial field)	Enhanced by interfacial field (OH ≥ 2.8 V)	PFOA t_1_/_2_ ~25 min (large droplets)	Medium, continuous generation at droplet surface	[74]

**Table 2:** Key Enzyme Systems and Gene Expression Profiles for the Biocatalytic Degradation of PFAS. ↑ indicates an increase or upregulation compared with the corresponding control or baseline condition.

Technology	Key Active Species	Changes in Functional Gene Expression	Enzyme–Substrate Interaction Mechanism	Refs.
Algal–bacterial granular sludge biosorption & co-metabolism	Algal–bacterial symbiotic system + functional microbial consortia	Denitrification gene abundance ↑ 16.1%; HAO gene expression ↑ 210.1%; 14 TCA cycle-related genes ↑	Electrostatic/hydrogen bonding between amide/hydroxyl groups in EPS and PFOA; hydrophobic interaction between hydrophobic groups and fluorocarbon chains	[29]
Photocatalytic pretreatment + fungal co-metabolism	Iodine-deficient BiOI photocatalyst + fungus *Cunninghamella elegans*	Upregulation of fungal CYP genes, avoiding inhibition by 5:3 FTCA	Photocatalytic generation of ·O_2_^−^ attacking PFOA carbon chain; fungal enzyme systems catalyzing reduction of PFOA to 6:2 FTOH	[75]
Microencapsulated enzymatic degradation	Dehalogenase & composite microbial consortia (*Paracoccus*, *Hyphomicrobium*)	High expression of methanol dehydrogenase & alkane degradation genes; *crcB* gene expression ↑ 2.3×	Dehalogenase binding to sulfonate group of PFOS, cleaving C–S bond; hydrophobic adsorption of PFOS by polysulfone membrane	[16]
Osmotic microbial fuel cell	Anode electroactive bacteria + biofilm microbiota	Stable expression of electroactive bacterial electron transfer genes; upregulation of EPS synthesis genes (*epsA*, *epsB*)	Electrostatic/hydrogen bonding & hydrophobic interaction between protein amide/hydroxyl groups in EPS and PFOA/PFOS	[76,77]
Microbial enzymatic degradation (bacterial degradation of PFAAs)	Dehalogenase, monooxygenase, etc. (*Acidimicrobium* sp. A6, *Pseudomonas*, etc.)	*rdhA* gene expression in *Acidimicrobium* sp. A6 positively correlated with F^−^ release; upregulation of *dhaA* & *dehH1* in *Pseudomonas mosselii*	Dehalogenase nucleophilic attack on C–F bond via conserved Asp-His-Asp catalytic triad; monooxygenase inserting oxygen into C–S bond	[17,18]
Anaerobic co-metabolic defluorination by *Acidimicrobium* sp. A6	*Acidimicrobium* sp. A6 + synergistic consortium	Expression of *rdhA* & *crcB* in A6 ↑ 2–3×; dehalogenase genes in *Paraburkholderia* positively correlated with F^−^ generation	Reductive dehalogenase attacking α/ε/δ C–F bonds of PFOA; linear PFAAs exhibiting higher binding affinity	[78]
Pure culture enzymatic degradation by *Pseudomonas*	*Pseudomonas aeruginosa*, *Pseudomonas putida*	Upregulation of defluorination gene (*dhaA*) & monooxygenase gene (*todC*)	Defluorination enzyme catalyzing C–F bond hydrolysis; monooxygenase activating substrate via hydroxylation	[19]

**Table 3:** Characteristics and Performance Comparison of PFAS Reductive Defluorination via Hydrated Electrons. ΔG‡ denotes the activation Gibbs free energy, and ‡ indicates the transition state.

Technology	Key Reactive Species	Reduction Potential	Electron Transfer Rate Constant	Defluorination Energy Barrier	Refs.
UV/VUV/Sulfite Photoreduction	e_a_q^−^	−2.9 V	1.8 × 10^6^–1.3 × 10^9^ M^−1^ s^−1^	C–F BDE: 106.8–485 kJ/mol	[71,80,81,82,83,84,85]
Plasma Reduction	e_a_q^−^, •OH, RNS, Energetic Electrons, etc.	−2.8 to −2.9 V	0.016–0.37 min^−1^	C–F BDE: 116–485 kJ/mol; C–Cl: 77.8 kcal/mol	[65,71,86,87,88,89]
Electrochemical Reduction	e_a_q^−^/Cathodic Electrons	−1.4 to −1.8 V vs. SHE	1.30 × 10^−4^–2.67 × 10^−4^ s^−1^; k_s_ = 0.0341 cm s^−1^	ΔG‡ = 2.43 eV; C–F BDE reduced by ~20%	[90,91]
Plasma-Cathode Electrochemical Reduction	Gaseous Electrons	~390 V	2.89 × 10^8^ m^4^ mol^−1^ s^−1^	C–F BDE: ~5 eV	[92]
Photocatalytic/Photoelectrocatalytic Reduction	Conduction Band Electrons (e_cb^−^)/Photogenerated Electrons	−0.76 to −0.99 V vs. SCE	0.2–1.53 h^−1^	C–F BDE: 400–550 kJ/mol; ΔG = −0.267 eV	[93,94,95]
Pd^0^-Biofilm Synergistic Reduction	H•, Dehydrogenase/Dehalogenase	−0.5 to −1.0 V vs. NHE	0.28 µM h^−1^	C–F BDE: 441 kJ/mol	[96]
Microwave Discharge Plasma-Fe^2+^ Synergistic Reduction	e_a_q^−^, OH	−2.9 V	9.9 × 10^−2^ min^−1^	C–F BDE: ~530 kJ/mol	[97]
UV/Zero-Valent Iron Photocatalytic Reduction	e_a_q^−^, Fe^2+^/Fe^3+^	−2.9 V	0.308–1.151 h^−1^	C–F BDE: ~485 kJ/mol	[98]
UV/DIHA Heterogeneous Photoreduction	Surface Electrons, •OH	−2.3 to −2.87 V vs. SHE	-	–CF_2_– BDE: 100–120 kcal/mol	[99]
Gas–Liquid Interface Spontaneous Reduction	e_a_q^−^, •OH	−2.9 V	-	C–F BDE: ~530 kJ/mol	[100]
Solar-Driven UV/Sulfite Reduction	e_a_q^−^, SO_3_•^−^	−2.9 V vs. SHE	~10^7^ M^−1^ s^−1^ (affected by DOM)	Barrier: 280–480 kJ/mol	[69]
DBD/Sulfite–Ultrafiltration Coupled Process	•OH, SO_4_•^−^, e_a_q^−^, ·O_2_^−^	-	-	e_a_q^−^ lowers C–F bond energy barrier	[101]

**Table 4:** Mechanisms and Kinetics of PFAS Degradation by Nonradical Pathways.

Technology	Key Active Species	Direct Electron Transfer/Degradation Kinetics	Refs.
Adsorption–Photocatalytic Synergy	Surface Holes (h^+^)	Pseudo-first-order rate constant (k) = 0.08–0.918 h^−1^	[105,106,107,108,109,110]
Electrochemical Direct Oxidation	Direct Electron Transfer	Pseudo-first-order k = 0.0175–0.946 min^−1^	[111,112,113,114,115]
Thermal Decomposition	Pyrolytic Action	Pseudo-first-order k = 0.02–0.14 min^−1^	[116,117,118,119,120,121,122]
Hydrothermal Degradation	Catalytic Decomposition + OH^−^ Nucleophilic Attack	Pseudo-first-order k = 0.0204 min^−1^ (for PFOA)	[123,124,125]

**Table 9:** TRL-based application-oriented assessment of PFAS degradation and supporting treatment technologies.

Technology	Representative Technologies	Practical Readiness	Energy Demand	Relative Cost	Most Realistic Application Window	Main Barriers	Refs.
Relatively close to implementation	Electrochemical oxidation, plasma treatment, thermal decomposition, supercritical water oxidation	Medium to high	High	High	Concentrated PFAS streams, spent adsorbents, regenerants, and industrial wastewater	High energy demand, electrode/reactor cost, long-term stability, mass-transfer limitation, byproduct control	[11,12,13,52,53,54,55,56,57,58,59,60,61,62,63,64,65,66,111,112,113,114,115,116,117,118,119,120,121,122,123,124,125]
Transitional technologies requiring pilot validation	UV/VUV/sulfite reduction, persulfate activation, sonochemical degradation, selected nonradical catalytic or electrochemical systems	Medium	Medium to high	Medium to high	Pretreated waters, concentrated streams, and hybrid treatment systems	Matrix quenching, reagent consumption, incomplete defluorination, limited continuous-flow and real-water data	[14,15,28,69,70,71,80,81,82,83,84,85,93,94,95,105,106,107,108,109,110,111,112,113,114,115]
Mainly laboratory-stage technologies	Biodegradation, enzymatic degradation, microdroplet reactions, many photocatalytic/photoelectrocatalytic systems	Low to medium	Low to medium	Low to medium at bench scale; uncertain at scale-up	Polishing steps, pretreatment-assisted systems, and niche applications	Slow kinetics, substrate specificity, idealized reaction conditions, limited mineralization evidence	[16,17,18,19,29,47,48,49,50,51,72,73,74,75,76,77,78,93,94,95,105,106,107,108,109,110]
Supporting/enabling separation technologies	Adsorption and ion exchange	High for separation, low for destruction alone	Low to medium	Medium; depends on media regeneration and disposal	Front-end enrichment before destructive treatment	Phase transfer rather than destruction, secondary waste generation, spent media disposal	[9,10]

## Data Availability

No new data were created or analyzed in this study. Data sharing is not applicable to this article.
